# Aluminum Complexation with Malate within the Root Apoplast Differs between Aluminum Resistant and Sensitive Wheat Lines

**DOI:** 10.3389/fpls.2017.01377

**Published:** 2017-08-03

**Authors:** Peter M. Kopittke, Brigid A. McKenna, Chithra Karunakaran, James J. Dynes, Zachary Arthur, Alessandra Gianoncelli, George Kourousias, Neal W. Menzies, Peter R. Ryan, Peng Wang, Kathryn Green, F. P. C. Blamey

**Affiliations:** ^1^School of Agriculture and Food Sciences, The University of Queensland, Brisbane QLD, Australia; ^2^Canadian Light Source Inc., Saskatoon SK, Canada; ^3^Elettra – Sincrotrone Trieste Trieste, Italy; ^4^CSIRO Agriculture and Food, Canberra ACT, Australia; ^5^College of Resources and Environmental Sciences, Nanjing Agricultural University Nanjing, China; ^6^Centre for Soil and Environmental Research, School of Agriculture and Food Sciences, The University of Queensland, Brisbane QLD, Australia; ^7^Centre for Microscopy and Microanalysis, The University of Queensland, Brisbane QLD, Australia

**Keywords:** aluminum toxicity, apoplast, distribution, malate, organic acids, speciation

## Abstract

In wheat (*Triticum aestivum*), it is commonly assumed that Al is detoxified by the release of organic anions into the rhizosphere, but it is also possible that detoxification occurs within the apoplast and symplast of the root itself. Using Al-resistant (ET8) and Al-sensitive (ES8) near-isogenic lines of wheat, we utilized traditional and synchrotron-based approaches to provide *in situ* analyses of the distribution and speciation of Al within root tissues. Some Al appeared to be complexed external to the root, in agreement with the common assumption. However, root apical tissues of ET8 accumulated four to six times more Al than ES8 when exposed to Al concentrations that reduce root elongation rate by 50% (3.5 μM Al for ES8 and 50 μM for ET8). Furthermore, *in situ* analyses of ET8 root tissues indicated the likely presence of Al-malate and other forms of Al, predominantly within the apoplast. To our knowledge, this is the first time that X-ray absorption near edge structure analyses have been used to examine the speciation of Al within plant tissues. The information obtained in the present study is important in developing an understanding of the underlying physiological mode of action for improved root growth in systems with elevated soluble Al.

## Introduction

Acid soils comprise ca. four billion ha of the global ice-free land or ca. 40% of the world’s arable land (von Uexküll and Mutert, 1995; Eswaran et al., 1997). In acid soils, the increased solubility of Al-containing minerals (Lindsay, 1979) increases Al^3+^ and Al-hydroxy anions that impact upon many cellular functions (Taylor, 1991). Root apices are the most sensitive part of the root (Ryan et al., 1993) and the apoplast is an important site for Al toxicity (Horst et al., 2010). Most cellular Al accumulates in the cell wall of the root apices due to the negative charges on the pectic and hemicellulose polysaccharides such as xyloglucans (Taylor et al., 2000; Yang et al., 2011; Zhu et al., 2012). As Al^3+^ binds to these charges, it stiffens cell walls thereby decreasing their loosening as required for cell elongation (Cosgrove, 1989; Jones et al., 2006; Kopittke et al., 2015). Accumulation of Al in the apoplast can be reduced by chemical changes that decrease charge density in the cell wall. For instance, transgenic plants engineered with greater pectin methylation or greater *O*-acetylation on the xyloglucan chains accumulate less Al and are more resistant of Al stress (Schmohl et al., 2000; Eticha et al., 2005a; Yang et al., 2008; Zhu et al., 2014). The importance of the apoplast in Al toxicity is highlighted by the Nramp aluminum transporter (NRAT1) in rice (*Oryza sativa* L.) (Xia et al., 2010). This transporter also reduces Al in the cell wall and increases Al resistance by vacuolar Al sequestration in roots cells. However, cell wall stiffening due to the accumulation of Al in the apoplast is not the only toxic interaction with Al^3+^ since application of Al^3+^ specifically to the elongation zone of maize caused considerable damage to the outer tissue layers but did not inhibit root growth (Ryan et al., 1993). Also, Al causes damage intracellularly by affecting Ca concentrations, interfering with cell division (Doncheva et al., 2005), disrupting the Golgi and mitochondrial functions (Bennet et al., 1985; Yamamoto et al., 2002), and compromising membrane integrity (Yamamoto et al., 2001).

Plant species that accumulate Al are able to tolerate high concentrations of Al within their tissues through production of organic acids (including oxalic acid and citric acid) which result in the formation of non-toxic Al-complexes (Ma et al., 1997a,b). In plant species such as wheat (*Triticum aestivum*) that do not accumulate Al in foliar tissues, it is proposed that Al is detoxified by the release of organic anions, particularly malate (Delhaize et al., 1993b; Ma et al., 2001) and citrate (Ryan et al., 2009; Garcia-Oliveira et al., 2014), into the rhizosphere which chelates Al thereby reducing its toxic effects within the root.

Delhaize et al. (1993b) showed that Al-induced release of malate is the major mechanism of Al resistance in wheat, with the secretion of malate from wheat roots occurring within 15 min of exposure to Al^3+^. Furthermore, malate excretion increases with increasing Al concentration and occurs largely from the apical 3–5 mm of the root. Much is already known about the release of malate by root apices (Delhaize et al., 1993b, 2007; Ryan et al., 1995a; Pellet et al., 1996; Ma et al., 2001; Kochian et al., 2004; de Andrade et al., 2011). However, Kinraide et al. (2005) noted that although many investigators have stated that Al resistance results from secretion of organic acid anions into the rhizosphere, there is not much data directly supporting this. Delhaize et al. (2007), furthermore, stated that the organic acid anions likely chelate Al either in the immediate vicinity of the apoplasm, or even within the apoplasm itself.

Some studies have reported that the concentration of malate in the root apex does not differ between Al-resistant and Al-sensitive near-isogenic lines (NILs) (the NILs differing in their Al-resistance due to the increased production of malate by the resistant NIL relative to the sensitive NIL) (Delhaize et al., 1993b; Ryan et al., 1995a) – this suggests that Al is not complexed by malate within the root tissues. Indeed, the complexation of Al within the root of the Al-resistant genotype would be expected to result in a higher malate concentration in these Al-resistant cultivars. However, such analyses assume that the selected analytical approach not only measures free malate, but also the Al-malate complex. By contrast, Tian et al. (2013), also using NILs of wheat, reported that malate in root apices is indeed higher in the Al-resistant genotype relative to the Al-sensitive genotype. Furthermore, when seedlings were exposed to concentrations of Al that result in the same inhibition of root elongation (5 μM for Al-sensitive genotype and 50 μM for Al-resistant genotype), Kikui et al. (2007) reported that two to three times more Al accumulated in the apical tissues of the Al-resistant genotype. This again suggests the possibility that Al is not only complexed external to the root but also within the root tissues.

To determine whether Al is complexed within the root itself or within the rhizosphere requires an assessment of both the speciation and distribution of Al within the root tissue. However, few studies have provided this information for roots of NILs or cultivars that differ in Al-resistance and organic acid production. Rather than examining the speciation of Al within root tissues, studies have generally examined the concentration of malate, either in solution or within the root tissue. Whilst the concentration of malate is likely to influence the speciation of Al, it is not a measure of Al speciation *per se*. The distribution of Al within root tissues is also important, with Delhaize et al. (1993a) utilizing energy-dispersive X-ray spectroscopy coupled with scanning electron microscopy (SEM-EDS) to examine the distribution of Al in roots of the two NILs of wheat, ES8 and ET8, for example.

The aim of the present study was to determine whether Al is present as an Al-malate complex within the root tissues and whether the complexation of Al by malate influences Al distribution. We do not question the importance of malate (and other organic anions) in enabling plants to resist elevated levels of Al, but rather, we aimed to investigate whether Al is complexed by malate externally to the root or within the root itself (or both). We used NILs of wheat, ES8 and ET8, which differ in alleles for TaALMT1 (the major gene for Al resistance that control malate efflux) with a resultant ca. 10-fold difference in their tolerance to Al in nutrient solution experiments. For these NILs, the speciation of Al within the root apices was analyzed *in situ* using synchrotron-based X-ray absorption near edge structure (XANES) spectroscopy. Changes in the cellular and sub-cellular distribution of Al were also analyzed *in situ* using synchrotron-based low energy X-ray fluorescence (LEXRF) microscopy. It is not the purpose of this study to further examine the kinetics or magnitude of malate production or secretion but rather to investigate the uncertainty of where the malate anion chelates Al and affords greatest protection: in the rhizosphere as suggested by Ma et al. (2001) and many others or within the root itself (defined here as being either within the apoplast or within the symplast). This study provides information regarding the underlying mechanism whereby organic acids confer resistance to excess Al in the rooting medium.

## Materials and Methods

### General Experimental Procedures

Solution culture experiments were conducted with two NILs, ES8 and ET8, that differ in Al resistance at a single locus, TaALMT1, with ET8 having greater malate efflux than ES8 in the presence of Al (Delhaize et al., 1993a). Seeds were placed in rolled paper towel suspended vertically in tap water in a laboratory maintained at 25°C (Kopittke et al., 2008). After 2 days, the seedlings were placed on top of a 600 mL beaker filled to the brim (650 mL) with 1 mM CaCl_2_ and 5 μM H_3_BO_3_ solution reduced to pH 4.6 using 0.1 M HCl. After ca. 18 h, the seedlings were transferred to the treatment solutions in which Al was added using appropriate volumes of a 10 mM AlCl_3_ stock solution. All solutions were adjusted to pH 4.6 and continuously aerated unless otherwise stated.

### Dose–Response Curves

A total of 13 treatments was prepared in 1 mM CaCl_2_ and 5 μM H_3_BO_3_, with seven Al concentrations for ES8 (0, 2.5, 3.5, 5, 10, 25, and 100 μM Al) and six for ET8 (0, 5, 10, 25, 50, and 100 μM). Each experimental unit consisted of three seedlings, with each treatment having three replicates. Except for the 100 μM treatment at pH 4.5, solutions were adjusted to pH 4.6 using 0.1 M HCl immediately following the addition of Al. Digital photography (Canon SX10IS) was used to allow measurement of root length (Kopittke et al., 2008), with images captured at the time of transfer to the Al-containing solutions (0 h), and after 6, 12, 18, 24, and 48 h. Root elongation rate (RER) was calculated following analysis of the images using ImageJ version 1.45s^1^.

### Bulk Al Concentration in Root Tissues

A total of 10 treatments were utilized for the measurement of bulk tissue Al concentrations, with roots of ES8 and ET8 exposed to 0, 3.5, and 50 μM Al for 3 and 48 h. Each experimental unit consisted of two replicates of 15 seedlings. After exposure to Al for the required periods, the roots were dipped in 1 mM CaCl_2_ for 1 min before the root apices (5 mm) were excised, excess moisture removed using filter paper, and weighed. Tissues were digested using a 5:1 mixture of nitric and perchloric acids before analysis for Al using inductively coupled plasma optical emission spectroscopy.

### Root Tissue Cation Exchange Capacity

After ca. 18 h growth in basal solutions, seedlings of ES8 and ET8 were transferred to new solutions containing 1 mM CaCl_2_ and 5 μM H_3_BO_3_ (with 0 μM Al) for a further 3 h. Each experimental unit consisted of two replicates of 70 seedlings. The cation exchange capacity (CEC) of the apical root tissues was measured using Cu-sorption (Blamey et al., 2014; Meychik et al., 2014). Briefly, seedlings were transferred for 1 h to a solution of 5 μM CuCl_2_ at 4°C and pH 4.6 to allow Cu sorption but limit metabolic changes. Adsorption of Cu binds rapidly and strongly to cell walls of roots (Kopittke et al., 2011) and hence provides an estimate of CEC.

### Cellular and Subcellular Distribution of Al in Root Tissues

The cellular and subcellular distribution of Al was assessed using synchrotron-based LEXRF (Kaulich et al., 2009) in an experiment of six treatments, with both ES8 and ET8 exposed to 3.5 μM Al for either 3 or 48 h, and ET8 also exposed to 50 μM Al for 3 or 48 h. After growth in Al-containing solutions for the appropriate length of time, 200-μm transverse sections were cut 3 mm from the apex, placed in planchettes filled with hexadecane, and frozen in a high pressure freezer (Bal-tec HPM010). The high pressure freezing was used to ensure rapid freezing (within milliseconds). Thereafter, the planchettes were split apart and stored under liquid nitrogen before freeze substitution (Leica EM AFS2) in 2% (v/v) glutaraldehyde in acetone at -90°C for 48 h, warming to 20°C, washing in ethanol, infiltration with LR White Resin, and polymerization. After storage at ambient temperature, a Reichert Ultracut Microtome was used to cut 5-μm thick sections and placed on 4-μm thick Ultralene Film. The sections were cut from within the middle of the 200-μm transverse sections in order to avoid any cells that were damaged during cutting of the fresh roots. Previous studies using this technique have shown that cellular contents (including the vacuoles) remain intact during this processing (Kopittke et al., 2015).

The LEXRF measurements were conducted at the TwinMic beamline (BL 1.1L) at ELETTRA, Italy (Gianoncelli et al., 2016) with eight Si-drift detectors in an annular back-scattering configuration positioned around the specimen (Gianoncelli et al., 2009). Selected regions were scanned with 1.7 keV excitation energy with a 0.7 μm step size (pixel) and a dwell time of 3–6 s per pixel (longer dwell times were used for samples in which tissue Al concentrations were expected to be lower). Each individual map was 60 μm × 60 μm (85 × 85 pixels) with scans taking 6–12 h to complete depending upon the dwell time. It was only possible to scan a small proportion of the root cross-sectional area given that the diameter of the root cylinder was ca. 500 μm. For all six samples, the area selected to be scanned (60 μm × 60 μm) focused on the rhizodermis and outer cortex – this being the area in which Al initially accumulates (Kopittke et al., 2015). The LEXRF spectra were fitted using PyMCA v4.7.3 (Sole et al., 2007).

### *In Situ* Assessment of Al Speciation in Root Tissues

The speciation of Al in root tissues was assessed using Al K-edge XANES spectroscopy. Seedlings were grown in solutions with Ca and B containing (i) 3.5 μM Al for 48 h (ES8), (ii) 50 μM Al for 3 h (ET8), and (iii) 50 μM Al for 48 h (ET8), yielding a total of three treatments, each with 30 seedlings. (We did not examine ES8 exposed to 3.5 μM for 3 h due to the low tissue Al concentration.)

Given that it is the apical 3–5 mm of the root which releases malate (Delhaize et al., 1993b), apical tissues (5 mm) were harvested, frozen in liquid nitrogen, and freeze-dried. The root apices from the 30 seedlings in each treatment were homogenized using a mortar and pestle at room temperature and spread evenly across a piece of double-sided carbon tape on a Cu holder. The samples were then analyzed in the X-ray absorption spectroscopy end-station at the SGM beamline of the Canadian Light Source (Saskatoon, SK, Canada) (Regier et al., 2007). The sample chamber was pumped to 10^-6^ Torr and spectra were acquired at the Al K-edge from 1,550 to 1,600 eV using a 10 s slew scan (Gillespie et al., 2015). The spectra presented are the average of 60 scans from different regions within each sample as measured in fluorescence with four silicon drift detectors. The spectra were normalized using the ion chamber (I0) spectrum collected simultaneously from an Au mesh in front of the sample and the energy scale calibrated using AlPO_4_ assuming a value of 1,566.1 eV.

Five reference standards were prepared for XANES analysis, being Al-malate, γ-Al_2_O_3_ (Alfa Aesar), gibbsite (reagent grade, synthetic, Wards Natural Science), AlPO_4_ (Sigma–Aldrich, 255963), and Al-pectin. The Al-malate was prepared using stock solutions with 50 mM AlCl_3_.6H_2_O and 200 mM L-malic acid. First, it was noted that a 25 mL solution with 20 mM Al and 100 mM L-malic acid (Sigma–Aldrich, 112577) had a pH of 1.9, and that 3.63 mL of 1 M NaOH was required to increase the pH to 4.5. Next, a new solution was prepared with the NaOH added to the L-malic acid prior to the addition of the Al – this being required to avoid the potential formation of highly toxic polymeric Al when alkali is added to Al-containing solutions (Bertsch and Parker, 1996). After mixing, the solution was frozen in liquid nitrogen and freeze-dried. Modeling with GeoChem-EZ (Shaff et al., 2010) indicated that >99.9% of the total Al was complexed with malic acid using the modified stability constants listed by Pellet et al. (1996). The Al-pectin standard was prepared using pectin from citrus fruit (Sigma–Aldrich, P9436). Sufficient KOH was added to achieve a negative charge of 38 μmol COO^-^/mL (McKenna et al., 2010), with a stock solution of 100 mM Al added to achieve 100% saturation. The Al-pectin gel was frozen in liquid nitrogen and freeze-dried.

## Results

### Effects of Al on Root Elongation Rate

**FIGURE 1:**
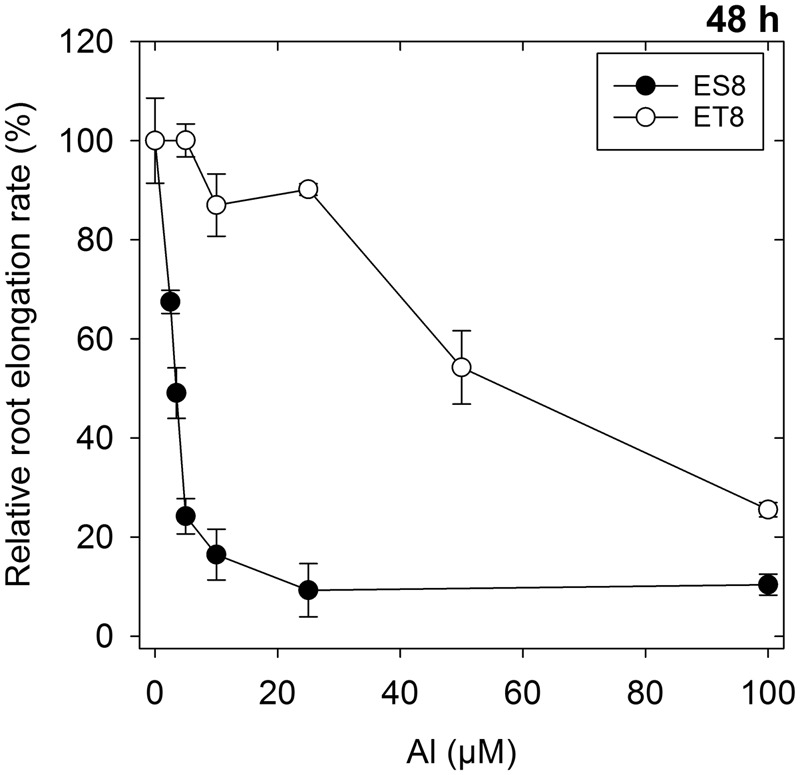
The effect of Al concentration on the relative root elongation rate of ES8 (Al-sensitive) and ET8 (Al-resistant) near-isogenic lines (NILs) of wheat. Roots were grown for 48 h in 1 mM Ca and 5 μM B solutions with 0 to 100 μM Al at pH 4.6. Data are the arithmetic means of three replicates (each with three seedlings) with the standard deviations shown.

**FIGURE 2:**
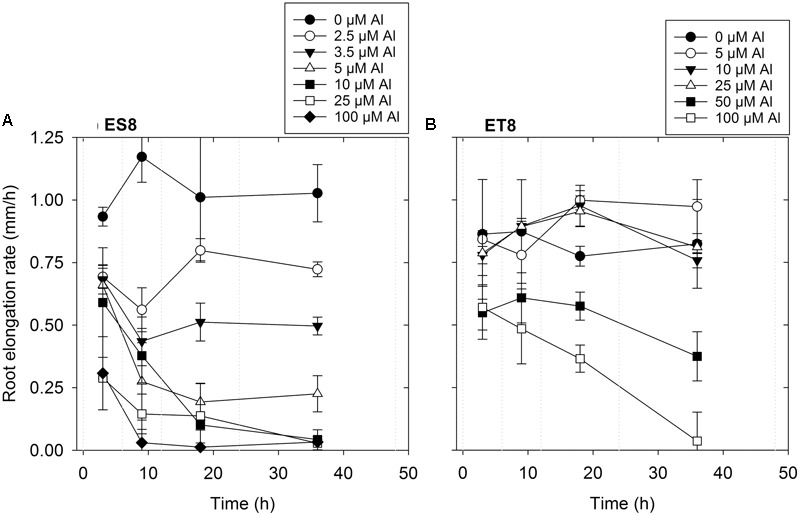
**(A,B)** Effects of Al on root elongation rate of two NILs of wheat, ES8 and ET8. Measurements of root length were made following exposure to Al for 0, 6, 12, 24, and 48 h (as indicated by the vertical dotted lines), with values for root elongation rate displayed mid-way through these periods.

**FIGURE 3:**
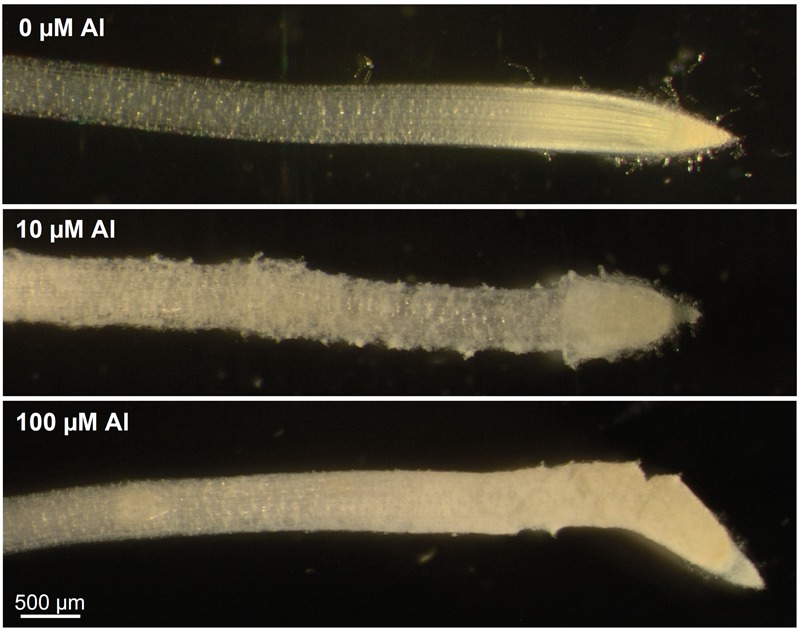
Light micrographs showing roots of ES8 exposed to 0, 10, or 100 μM Al for 48 h. Note the tearing and rupturing evident in the outer tissues of roots exposed to 10 or 100 μM Al. The scale bar applies to all three images.

Increased Al concentration in solution decreased RER of wheat seedlings, with the pattern of the response differing markedly between ES8 and ET8 as found by Kikui et al. (2007). Over 48 h, a 50% decrease in RER of ES8 occurred at 3.5 μM Al compared to a concentration of 50 μM Al for ET8, 14-times higher than for ES8 (**Figure 1**). It is noteworthy that these concentrations caused a slight reduction in RER of both NILs after only 6 h (**Figure 2**). The average RER of ES8 after 6 h exposure to 3.5 μM Al was 0.68 mm/h compared to 0.93 mm/h for the control; corresponding values for ET8 at 50 μM Al for 6 h were 0.55 and 0.86 mm/h. For ES8, in particular, exposure to Al in some treatments resulted in the rupturing and tearing of the outer walls of the rhizodermis (**Figure 3**).

Six treatments were identified for further investigation: (i, ii) roots of ES8 were exposed for 3 or 48 h to 3.5 μM Al (being sufficient to reduce RER slightly after 6 h and by 50% after 48 h), (iii, iv) roots of ET8 exposed for 3 or 48 h to 50 μM Al (being sufficient to reduce RER slightly after 6 h and by 50% after 48 h), and (v, vi) roots of ET8 exposed for 3 or 48 h to 3.5 μM Al (a concentration that did not decrease the RER of ET8 but decreased the RER of ES8 by 50% after 48 h). When selecting these treatments, it was noted that the excretion of malate begins rapidly and without any detectable delay (Delhaize et al., 1993b; Osawa and Matsumoto, 2001).

### Bulk Concentration of Al in Apical Root Tissues and Root Cation Exchange Capacity

**FIGURE 4:**
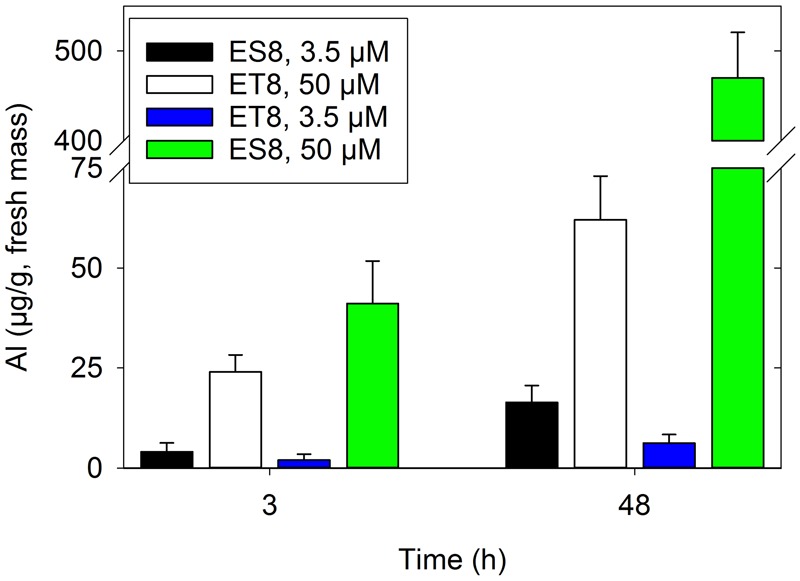
The concentration of Al in the root apical tissues (0–5 mm) of two wheat NILs grown for 3 and 48 h at either 3.5 or 50 μM Al. The concentrations resulting in a 50% reduction in root elongation rate are 3.5 μM Al for ES8 and 50 μM Al for ET8 (**Figure 1**). Tissue concentrations are presented on a fresh mass basis.

When grown in solutions containing Al at a concentration that decreased RER by 50% over 48 h (i.e., 3.5 μM Al for ES8 and 50 μM Al for ET8), Al in the fresh root apical tissues (i.e., both symplast and apoplast) was ca. four to six times higher for ET8 than for ES8 (**Figures 1, 4**). However, when ES8 and ET8 were grown at the same Al concentration, Al in the root apical tissues was substantially higher for ES8 than for ET8 (**Figure 4**). For example, when grown in solutions containing 50 μM Al, the root tissue Al concentration was 470 μg/g in ES8 but only 62 μg/g in ET8, with RER being reduced ca. 90% in ES8 compared to 50% in ET8.

The observation that the Al concentration was four to six times higher for ET8 than for ES8 (**Figure 4**) despite the same magnitude of reduction in RER (**Figure 1**) cannot be attributed to greater CEC in the apical root tissues of ET8 because measurements of CEC using Cu sorption indicated that the CEC of the root apices was similar in both ES8 and ET8 (0.78 ± 0.073 mmol_+_/kg for ES8 and 0.76 ± 0.073 mmol_+_/kg for ET8 on a fresh mass basis, with standard deviations).

### Lateral Distribution of Al in Root Tissues

**FIGURE 5:**
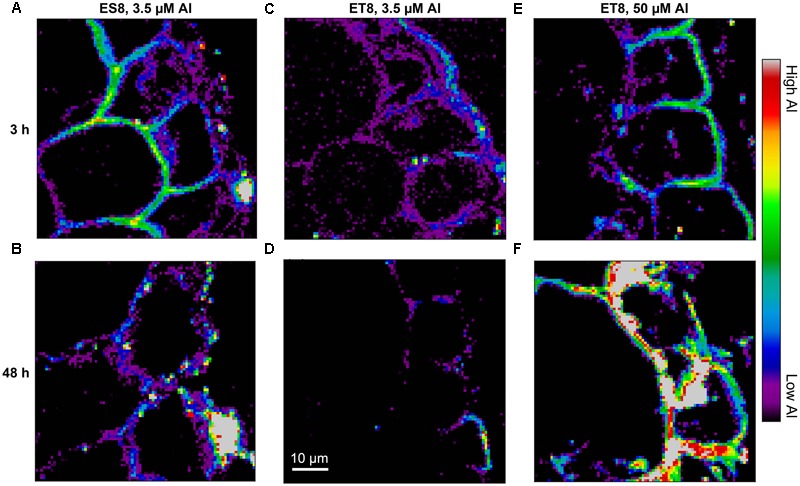
The distribution of Al, examined using LEXRF, in 5-μm-thick transverse root sections taken 3 mm from the apex of ES8 and ET8 exposed to 3.5 or 50 μM Al for either 3 h **(A,C,E)** or 48 h **(B,D,F)**. In all cases, the rhizodermis (and exterior of the root) is on the right-hand side of the image, with only the rhizodermis and 1–2 layers of cortical cells shown. The signal intensity is presented as a color scale, with brighter colors corresponding to higher concentrations. All images were scaled to the same values, and hence intensities can be compared between images. The scale-bar in **(D)** applies to all images. See Supplementary Figure 1 for corresponding light micrographs.

In all six treatments, Al as measured by synchrotron-based LEXRF was highest in the external root tissues, with the concentration decreasing from the rhizodermis through the outer to the inner cortex (**Figure 5** and Supplementary Figure 1). Furthermore, Al accumulated primarily in the cell wall, with comparatively small amounts of Al within the symplast. Of particular interest is the comparison (**Figures 5A,B,E,F**) between ES8 and ET8 at Al concentrations causing a similar reduction in growth (i.e., ES8 at 3.5 μM Al and ET8 at 50 μM Al). It is noteworthy that the distribution of Al within the rhizodermis and outer cortex was similar – most Al being located within the cell wall in all instances. We also compared ES8 with ET8 when both were grown at 3.5 μM – although causing a 50% reduction in RER of ES8, this Al concentration does not reduce RER of ET8. Again, the distribution of Al was similar in all four treatments, with most Al accumulating within the cell wall (**Figures 5A–D**). However, concentrations of Al in the walls of the rhizodermis and outer cortex were lower for ET8 than for ES8 exposed to 3.5 μM Al, both after 3 and 48 h exposure (**Figures 5A–D**).

### *In Situ* Analyses of Al Speciation in Root Apices

**FIGURE 6:**
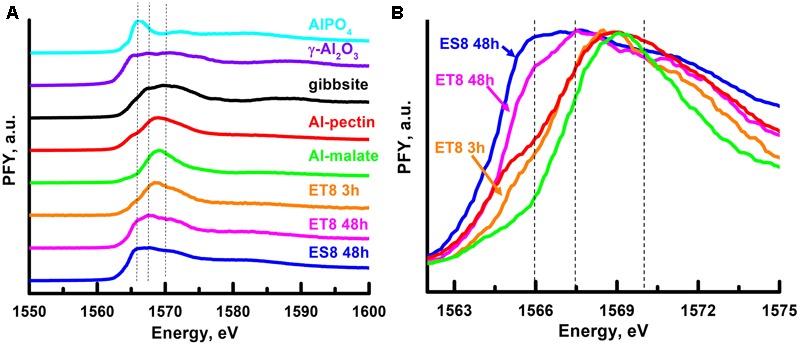
*In situ* analyses of Al speciation using synchrotron-based X-ray absorption near edge structure (XANES). **(A)** Al K-edge XANES spectra of five standard compounds and for root tissues of the near-isogenic wheat lines where ET8 was exposed to 50 μM Al for 3 or 48 h and ES8 was exposed to 3.5 μM Al for 48 h. **(B)** Enlarged spectra (1,562–1,575 eV) for the three root tissues and two standards of interest, Al-malate and Al-pectin. The dotted lines are provided for reference, being 1,566 eV (corresponding to the strong single maximum of four-fold coordinated Al, such as for Al-phosphate), 1,567.7 eV (corresponding to the six-fold coordinated peak of gibbsite), and 1,570 eV (corresponding to the six-fold coordinated peak of γ-Al_2_O_3_).

Differences were evident between the Al K-edge XANES spectra of the five standard compounds (**Figure 6**) with differentiation between six- and four-fold compounds as defined by Ildefonse et al. (1998). Specifically, the spectra of compounds with six-fold coordination environments generally have maxima at ca. 1,568 and 1,572 eV and sometimes have further features at higher energies, while those with four-fold coordination have a strong single maximum at ca. 1,566 eV and only weak features at higher energy. Accordingly, Al-phosphate (four-fold coordinated) had a distinct white-line peak at ca. 1,566 eV and gibbsite (six-fold coordinated) had distinct peaks at 1,567.7 and 1,570.5 eV. With coordination numbers of 4 and 6, γ-Al_2_O_3_ had distinct peaks at 1,565.8, 1,567.1, and 1,570.5 eV.

The Al-malate standard was utilized to examine effects of low molecular weight organic acids commonly produced by plant roots, including those of wheat. The Al-malate standard prepared in this study, with a malate/Al molar ratio (MR) = 5, had a single sharp peak at 1,569.1 eV, indicating six-fold coordination. The crystalline Al-malate compounds (malate/Al MR = 1 or 2) investigated by Happel et al. (2007) were also observed to have six-fold coordinated Al. In these cases, the Al_4_O_6_-core was the main structural element, with each Al coordinated to at least one malate molecule. In another study (Xu et al., 2010), precipitates prepared from solutions with malate/Al MR = 0.001, 0.01 and 0.1 consisted of a mixture of crystalline Al (oxy)hydroxides and short-ranged ordered (SRO) Al-malates. In this study of Xu et al. (2010), the Al K-edge spectra of the malate/Al MR = 0.001 and 0.01 precipitates were similar to that of the crystalline Al (oxy)hydroxides, masking any contribution from the SRO Al-malates. However, the Al K-edge spectra of the malate/Al MR = 0.1 precipitate contained two broad peaks at 1,565.9 eV (four-fold coordination) and 1,570.4 eV (i.e., six-fold coordination). Similar to the Al-malate precipitate prepared in the present study, Xu et al. (2010) found only one maximum for the six-fold coordinated Al in the malate/Al MR = 0.1 system, suggesting that most of the Al was bonded to at least one malate molecule. There were also significant amounts of four-fold coordinated Al as the intensity of the four-fold peak was similar to that of the six-fold peak. Regardless, these results support our hypothesis that most, if not all, of the Al in the present study was coordinated to malate, given that there was only a single peak in the Al K-edge spectrum for the six-fold coordinated Al (**Figure 6**). Note that for ‘pure’ Al-(O_3_PC_6_H_5_) complexes (aluminophosphonate), where all Al is bonded to at least one -O_3_PC_6_H_5_ ligand, there are two peaks in the Al K-edge spectrum for the six-fold coordinated Al, suggesting that only when C is in the second shell there is not splitting of the six-fold coordinated peak (Chaplais et al., 2001).

A pectin standard was also investigated because polysaccharides are reported to be the main site for Al sorption in roots (Taylor et al., 2000; Horst et al., 2010). The Al K-edge spectrum for the Al-pectin compound had a low intensity peak at 1,565.3 eV (four-fold coordination) and a single peak at 1,569.0 eV (consistent with a six-fold coordination) that was broader than that for the Al-malate spectrum (**Figure 6**). The amount of four-fold coordinated Al was much less than that of the six-fold coordinated Al as is evident from the weaker intensity of the four-fold (1,565.3 eV) versus six-fold coordinated peak (1,569.0 eV). Thus, it appears that most of the Al was coordinated to at least one pectin molecule.

Next, the Al K-edge XANES spectra of the three root apical samples were examined (**Figure 6**). Firstly, it should be noted that the spectra from the root samples are not identical to those of the reference compounds, indicating a different Al environment in the root samples from that of the reference compounds and that Al coordination is perhaps present as a mixture. Secondly, it was noted that there were clear differences between the spectra of the three samples, depending upon both the time of exposure (comparing ET8 exposed for 3 or 48 h) and between NILs (comparing ES8 and ET8 exposed for 48 h) (**Figure 6B**). For apices of ET8 exposed for 3 h, the spectrum consisted of a broad peak at 1,568.6 eV (six-fold coordination) with distorted symmetry at higher energy compared to the Al-malate spectrum, and a small peak at 1,565.6 eV (four-fold coordination) with lower intensity than that observed in the Al-pectin spectrum (**Figure 6**). The six-fold coordinated Al peak was ca. 0.5 eV lower compared to that of the Al-malate and Al-pectin six-fold coordinated Al peak. Thus, it is likely that the Al in the apices of ET8 exposed for 3 h is a mixture of Al species, consisting mainly of Al organic species, including that of Al-malate, Al-pectin, and perhaps a small contribution from inorganic Al species. For apices of ET8 exposed for 48 h, the spectrum consisted of two peaks at 1,567.7 and 1,570.3 eV (coordination number of 6) and a peak at 1,566.1 eV (coordination number of 4). The presence of a better resolved peak in the ET8 48 h spectrum compared to the ET8 3 h suggests that there was a slight increase in the inorganic Al species with time. The six-fold maxima occurred nearly 1 eV lower compared to that observed in the ET8 3 h spectrum, suggesting that the organic species coordinated to Al were likely changing with time. This could potentially be due to increased polymerization of the organics or Al (Hu et al., 2008). The intensity of the four-fold coordinated Al peak was nearly equal that of the 1,567.7 eV peak, indicating that there was considerably more four-fold coordinated Al in the ET8 48 h tissues compared to the ET8 3 h and Al-pectin standard. The spectrum for root apical tissues of ES8 exposed for 48 h was similar to that of ET8 48 h except that the intensity of the four-fold coordinated peak was similar to that of the six-fold maxima in the ET8 48 h spectrum (**Figure 6**). Thus, in summary, XANES analyses indicated clear differences between the three root tissue samples, with Al in the apices of ET8 exposed for 3 h being a mixture of Al species, but mainly Al organic species including that of Al-malate and Al-pectin.

## Discussion

There is conclusive evidence that organic acid secretion by many plants, including wheat, mitigates the toxic effects of soluble Al by forming harmless complexes (Ma et al., 2001; Ryan et al., 2001; Kochian et al., 2004). However, uncertainty remains as to what extent these complexes form within the root tissue (apoplast and symplast) or in the rhizosphere. We provide evidence that both these processes occur. We utilized synchrotron-based LEXRF to show that Al (including Al-malate complexes) accumulates largely within the apoplast – this being the dominant compartment of Al-accumulation regardless of treatment. The presence of Al-malate complexes within apoplast protects the root by limiting the binding of Al to the negatively charged cell wall components which reduces cell elongation and other processes (Horst et al., 2010; Kopittke et al., 2015). It is anticipated that this study will assist in improving plant growth in acid soils high in soluble Al by improved understanding of the physiological mechanisms of Al resistance.

### Al Complexation External to the Root

Consistent with studies comparing sensitive and resistant cultivars of wheat (Rincón and Gonzales, 1992; Delhaize et al., 1993b; Kikui et al., 2007), ET8 accumulated substantially less Al in the apical root tissues (apoplast and symplast) than did ES8 when grown at the same Al concentration in solution (**Figure 4**). This was not due to differences in the CEC of root apical tissues since these were similar at 0.78 mmol_+_/kg for ES8 and 0.76 mmol_+_/kg for ET8. Rather, the data indirectly confirms the importance of malate secretion into the rhizosphere where it complexes Al causing a substantial reduction in the entry of Al into the root tissue.

### Al Complexation within the Root

Not only is Al complexed by malate external to ET8 roots (as shown by decreased tissue concentrations when grown at the same Al in the rooting medium as ES8), some of the Al within the root itself is complexed by malate – this being evidenced by two observations. Firstly, when ES8 and ET8 were exposed to sufficient Al to reduce RER by 50% over 48 h (3.5 μM Al for ES8 and 50 μM for ET8, **Figure 1**), the apical root tissues of ET8 accumulated four to six times more Al than did those of ES8 (**Figure 4**). This difference in Al-accumulation could not be attributed to differences in the negative charge of the root tissues. This finding is similar to that reported by Kikui et al. (2007), who found that ET8 accumulated two to three times more Al in apical (10 mm) root tissues than did ES8 when grown at differing Al concentrations resulting in a comparable reduction in RER. Secondly, *in situ* synchrotron-based XANES analysis showed that Al was six-fold coordinated within 5 mm apical tissues of ET8 roots exposed to Al for 3 h, with the relatively sharp peak in the XANES spectrum (1,568.6 eV) indicating the likely presence of Al-malate together with other six-fold coordinated Al such as Al-pectin (**Figure 6**). After exposure to Al for 48 h, much of the Al was four-fold coordinated (particularly for ES8, with ET8 still having a higher proportion of six-fold coordinated Al), although the exact form of this four-fold coordinated Al is not clear and further work is required. To our knowledge, this is the first time that XANES analyses have been used to examine the speciation of Al within plant tissues (see review by Kopittke et al., 2016).

Given that Al-malate complexes were present within the root tissues, we utilized synchrotron-based LEXRF to examine the distribution of Al in transverse sections cut 3 mm from the apex. In contrast to morin, a fluorochrome that forms a fluorescent complex with Al (Eticha et al., 2005b), for example, LEXRF is able to detect all Al within plant tissues, including Al bound to the cell wall. In accordance with measurements of bulk concentrations (**Figure 4**), the LEXRF analyses indicated that when ES8 and ET8 were grown at 3.5 μM Al, roots of ES8 accumulated more Al than did those of ET8 (as is evident when comparing **Figures 5A,B** with **Figures 5C,D**). However, when grown at Al concentrations that resulted in the same reduction in RER, root tissues of ET8 tended to accumulate more Al than did those of ES8 (**Figure 4**, and compare **Figures 5A,B** with **Figures 5E,F**). The concentration of Al was highest in the rhizodermis in all six treatments, decreasing in the outer cortical tissues and even more so in the inner cortex. Furthermore, Al accumulated primarily in the cell wall in apical root tissues of both ES8 and ET8, with comparatively small amounts of Al found within the symplast (**Figure 5**) as found by Taylor et al. (2000). This indicates that Al complexed by malate within the root tissues of ET8 accumulated primarily within the apoplast of the rhizodermis and outer cortex.

It is noteworthy that roots, particularly for ES8, ruptured when exposed to high concentrations of Al (**Figure 3**) – this having been observed previously in the roots of a wide range of plant species (Ryan et al., 1993; Yamamoto et al., 2001; Kopittke et al., 2008; Motoda et al., 2010; Osawa et al., 2011). These ruptures form initially in the elongation zone due to the “differential expansion between Al-arrested epidermis cells and (the) still-expanding cortex cells” (Osawa et al., 2011). Interestingly, it is in these that are susceptible to rupturing (i.e., the rhizodermis and outer cortex) that Al was found to accumulate to high concentrations (**Figure 5**). This reaffirms the importance of Al accumulation in the cell walls, thereby decreasing root elongation by rapidly inhibiting the ability of the walls to loosen (Kopittke et al., 2015). Thus, it is apparent that the complexation of Al by malate in ET8, both in the rhizosphere and apoplast, reduces the strong binding of Al to the cell wall, and thereby reduces the damaging interactions of Al with the root cells.

The observation in the present study that Al is complexed by malate within the root tissues is in agreement with the theoretical modeling of Kinraide et al. (2005) who concluded that resistance to Al could not result solely from the secretion of malate into the rhizosphere with a concomitant reduction in Al^3+^ activity at the root surface. Our findings are also supported by those of Kikui et al. (2007) that much of the Al in the apical 0–4 mm of ET8 roots (but not of ES8 roots) was removed by rinsing with citrate, suggesting that Al in root tissues of ET8 is not bound strongly to the cell wall. Additionally, Zheng et al. (2004) concluded that the production of organic acids and subsequent formation of Al-organic complexes would likely reduce the binding strength of Al within the apoplast.

Finally, we can make some conclusion about whether the Al-malate complexes in root tissues are formed *in planta* or whether they are formed in the rhizosphere first and then penetrate the tissue. We know that the Al concentrations in ES8 and ET8 tissues are markedly higher in solutions containing uncomplexed Al than when Al is complexed with malate. For example, Kikui et al. (2007) reported that root apical tissues of ES8 exposed to 5 μM Al contained 0.31 nmol Al/apex but decreased to 0.15 nmol Al/apex when exposed to 50 μM Al with 100 μM malate (i.e., Al in the apex was halved despite a 10-fold increase in solution Al). We also showed here that the ES8 roots accumulate more Al than ET8 roots when exposed to the same Al concentration. Presumably this marked decrease in the presence of malate in ET8 occurs because the Al triggers the release of malate from the ET8 roots. While the trivalent Al^3+^ binds strongly to cell walls and hence accumulates rapidly in ES8 roots, the Al-malate complex does not. Tian et al. (2014) have shown similar results as have Blamey et al. (1997) with pectic complexes. In the present study also, root apical tissues of ET8 contained four-fold to six-fold more Al than did those of ES8 when exposed to Al concentrations causing a 50% reduction in RER (**Figure 4**) suggesting that the Al-malate complexes are formed *in planta*, rather than being taken up from the rhizosphere. However, further studies are required to test this hypothesis. Regardless of whether the Al-malate complexes within the apoplast form within the root or within the rhizosphere, it is known that Al-malate complexes are non-toxic (or at the least, substantially less toxic) than is the free Al^3+^ ion (Kerven et al., 1991; Ryan et al., 1995b; Pellet et al., 1996). In addition, previous studies have reported that organic acids (such as citrate or malate) are able to desorb Al from cell walls (Zhang and Taylor, 1990). This suggests that the release of malate into the apoplast could possibly detoxify apoplastic Al, even after it had initially bound to the cell wall.

## Conclusion

Although it is commonly assumed that Al is detoxified by the release of organic anions (such as malate) into the rhizosphere, the present study has shown that a substantial amount of the Al within root apices of the Al-resistant wheat NIL, ET8, is present as Al-malate complexes. We utilized synchrotron-based XANES to provide *in situ* information regarding the speciation of Al within apical root tissues. Furthermore, synchrotron-based LEXRF analyses demonstrated that Al (including any Al-malate complexes) accumulated predominantly within the apoplast of the rhizodermis and outer cortex thereby limiting the strong binding of Al^3+^ to cell wall components. The information obtained in the present study is important in developing an understanding of the underlying physiological mode of action whereby organic anions allow for improved growth in Al-toxic systems.

## Author Contributions

PK, NM, PR, and PW conceived the research program; PK, BM, and KG conducted the plant-growth experiments; PK, BM, AG, GK, and FB conducted LEXRF analyses at Elettra (Italy); PK, BM, CK, JD, and ZA conducted the analyses at the Canadian Light Source; PK, BM, PW, and FB carried out the data analyses; PK wrote the first draft of the article to which all other authors contributed.

## Conflict of Interest Statement

The authors declare that the research was conducted in the absence of any commercial or financial relationships that could be construed as a potential conflict of interest. The reviewer AATJ and handling Editor declared their shared affiliation, and the handling Editor states that the process nevertheless met the standards of a fair and objective review.

## Footnotes

1 http://imagej.nih.gov/ij/
