# Supplementary material for: Aluminum Complexation with Malate within the Root Apoplast Differs between Aluminum Resistant and Sensitive Wheat Lines
Source: Front Plant Sci. 2017 Aug 3;8:1377. doi: 10.3389/fpls.2017.01377 (PMC5541250; doi:10.3389/fpls.2017.01377)
Supplement: Supplementary file 1 [file Image_1.PDF]

frontiers

Supplementary Material

Aluminum Complexation With Malate Within The Root Apoplast Differs Between Aluminum Resistant And Sensitive Wheat Lines

Peter M. Kopittke, Brigid A. McKenna, Chithra Karunakaran, James J. Dynes, Zachary Arthur, Alessandra Gianoncelli, George Kourousias, Neal W. Menzies, Peter R. Ryan, Peng Wang*, Kathryn Green, F. Pax C. Blamey

*** Correspondence:** Peng Wang: p.wang3@njau.edu.cn

1 Supplementary Figures and Tables

1.1 Supplementary Figures

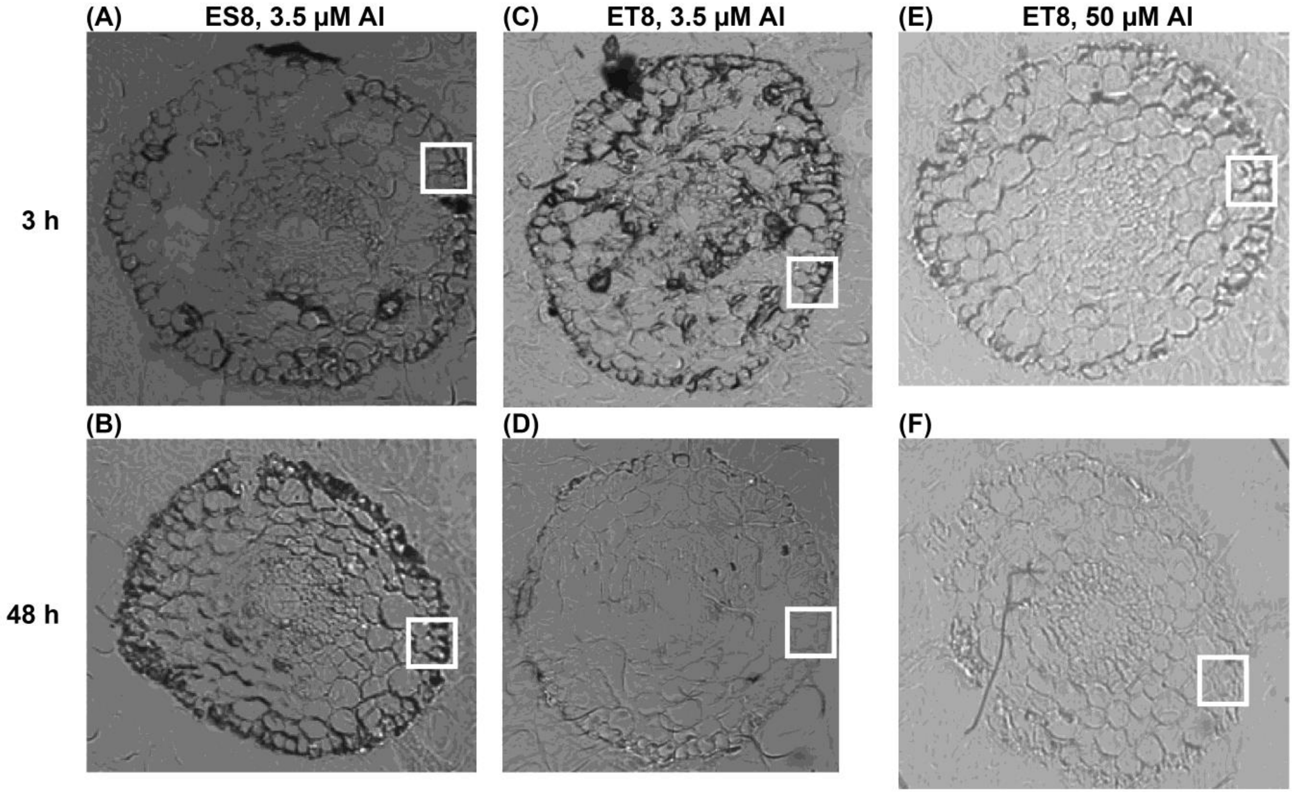

Supplementary Figure 1. Light micrographs of 5-μm-thick transverse root sections taken 3 mm from the apex of ES8 and ET8 exposed to 3.5 or 50 μM Al for either 3 h (A,C,E) or 48 h (B,D,F). The areas indicated by the white rectangles were analyzed using LEXRF (Figure 5 of main text).
